# Polyphony: an Interactive Transfer Learning Framework for Single-Cell Data Analysis

**DOI:** 10.1109/TVCG.2022.3209408

**Published:** 2022-12-20

**Authors:** Furui Cheng, Mark S Keller, Huamin Qu, Nils Gehlenborg, Qianwen Wang

**Affiliations:** Hong Kong University of Science and Technology.; Harvard University.; Hong Kong University of Science and Technology.; Harvard University.; Harvard University.

**Keywords:** Interactive Machine Learning, Transfer Learning, Single-cell Data Analysis, Human-AI Interaction

## Abstract

Reference-based cell-type annotation can significantly reduce time and effort in single-cell analysis by transferring labels from a previously-annotated dataset to a new dataset. However, label transfer by end-to-end computational methods is challenging due to the entanglement of technical (*e.g.*, from different sequencing batches or techniques) and biological (*e.g.*, from different cellular microenvironments) variations, only the first of which must be removed. To address this issue, we propose *Polyphony*, an interactive transfer learning (ITL) framework, to complement biologists’ knowledge with advanced computational methods. *Polyphony* is motivated and guided by domain experts’ needs for a controllable, interactive, and algorithm-assisted annotation process, identified through interviews with seven biologists. We introduce anchors, *i.e.*, analogous cell populations across datasets, as a paradigm to explain the computational process and collect user feedback for model improvement. We further design a set of visualizations and interactions to empower users to add, delete, or modify anchors, resulting in refined cell type annotations. The effectiveness of this approach is demonstrated through quantitative experiments, two hypothetical use cases, and interviews with two biologists. The results show that our anchor-based ITL method takes advantage of both human and machine intelligence in annotating massive single-cell datasets.

## 1 Introduction

The last decade has witnessed great progress in the area of single-cell omics technologies, which allow researchers to profile individual cells from complex tissues in increasingly efficient ways. Substantial efforts have been made in the Human Cell Atlas (HCA) Project [48], the Human Tumor Atlas Network (HTAN) [51], and the Human BioMolecular Atlas Program (HuBMAP) [24] to study human tissues at the single-cell level to create large-scale datasets, often referred to as single-cell atlases. These data provide promising opportunities to systematically understand complex biological processes at the cellular level, from discerning transitions of bone marrow cells, to understanding immune cell response to SARS-CoV-2 infection [26].

With the availability of single-cell atlases, it is of growing interest to molecular biologists to leverage the high-quality labeled datasets as references to annotate the sequencing results from new studies. Compared with conventional methods of using unsupervised clustering algorithms and manually labeling cell populations, reference-based methods are more efficient and reproducible. However, despite its greater efficiency, reference-based annotation can be challenging due to the technical variation (*e.g.*, different laboratory conditions) among different studies, referred to as batch effects in molecular biology literature [29] (Fig. 2). The results from different studies can be systematically biased, making it impossible to directly apply a supervised annotation model trained on the reference dataset to predict labels for the query dataset (unlabeled results from a new study). To make the problem more challenging, true biological variation (*e.g.*, in the form of unique cell populations that are only present in some samples but not in others) often exists among different studies and must be retained when removing batch effects. To achieve accurate reference-based annotation, a fundamental task is to integrate the reference dataset with a query dataset by learning a joint embedding space, where batch effects are removed while biological variation is preserved.

Existing work in bioinformatics and artificial intelligence domains focuses on designing computational and machine learning methods to solve this problem. For example, Symphony by Kang *et al.* [26], uses a linear mixed model to update the embedding of cells from the query dataset to fit the reference. ScArches by Lotfollahi *et al.* [37] is a transfer-learning framework that adapts the model trained on the reference dataset to the query dataset. However, computational methods are usually based on certain assumptions that do not always hold. For example, existing methods typically assume that query datasets share similar cell types and cell type distributions with the reference dataset, making them unstable for identifying novel cell populations in the query dataset. To conduct accurate and reliable reference-based annotation, it is important to involve biologists and integrate their domain knowledge into the annotation process, examining, refining, updating, and validating the automatic annotations and their underlying assumptions. Therefore, an interactive learning framework and a set of suitable interactive visualizations are needed.

Single-cell data analysis heavily relies on interactive visualizations [8]. A large number of interactive visualization tools are widely used in the community of single-cell analysis, such as Vitessce [28], CellxGene [9], Pagoda2 [3], and UCSC Cell Browser [59]. However, existing visualization tools are primarily designed for manually exploring and annotating individual datasets, with limited support for examining and modifying multiple datasets or reference-based annotation models. While valuable lessons can be learned from existing single-cell visualization tools, the set of visualizations and interactions required to facilitate human-AI collaboration in single-cell annotation has not been well explored.

In this work, we propose *Polyphony*, an interactive visualization approach to make the reference-query integration and label transfer process *understandable, interactive*, and *controllable*. The design and development of *Polyphony* have been driven and guided by multiple rounds of interviews with a total of seven experts in single-cell analysis. Core to *Polyphony* is an interactive transfer learning (ITL) framework and a set of interactive visualizations that support user-involvement in the ITL process. Specifically, the ITL framework extends scArches [37] to enable human interpretation and participation through anchors, analogous cell populations across datasets, a concept that is understood by both the back-end ITL model and human domain experts. For the back-end model, anchors guide the integration of different datasets, help remove batch effects, and enable more accurate label transfer and cell annotation. For human users, anchors are easily interpreted by comparing their biological markers such as gene expression levels. As shown in Fig. 3, *Polyphony* presents potential anchors to users through an *anchor recommender* and fine-tunes the back-end model through an *anchor processor* using anchors that are confirmed, refined, or added by users. We propose three coordinated views to empower users to interpret and interact with anchors by considering multiple types of information, including the structure of the embedding space, cell clusters, top differentially-expressed genes, and per-cell gene expression values. We conduct a quantitative experiment to evaluate the effectiveness of the *Polyphony* model by simulating different strategies that experts may apply to confirm anchors during the model iteration. We further introduce two use cases of *Polyphony* and report feedback from two individual interviews with experts to demonstrate the effectiveness of our approach. The results show that the anchor-based ITL method helps users to conduct reference-based joint analysis of single-cell datasets to obtain more accurate and trustworthy integration results.

The major contributions of this paper are:

- An abstraction of the domain problem and analysis tasks in reference-based single-cell data analysis.
- *Polyphony*, a visual analytics tool that tightly integrates interactive visualizations with an anchor-based interactive transfer learning framework to facilitate human-AI collaboration during single-cell data integration and annotation.
- A hybrid evaluation consisting of quantitative experiments, two use cases and feedback from experts demonstrating the usefulness of the system and inspiring future research directions.

## 2 Abstraction

### Domain Problem.

Single-cell transcriptomics examines the gene expression levels of thousands to millions of individual cells to understand cellular heterogeneity, reconstruct developmental trajectories, and model transcriptional dynamics. In this field, cell type identification is considered the most elementary analysis task, for which a large number of computational methods have been developed [32].

Reference-based annotation enables efficient cell type identification by transferring labels from reference datasets to new datasets. A critical step in the reference-based annotation is to integrate different datasets into the same embedding space, which allows labels to be transferred from one dataset to another. Batch effects occur when non-biological factors (*e.g.*, sequencing instrument differences) result in systematic biases in the data produced by an experiment. Batch effects can be difficult to resolve since they are usually coupled with biological variability among different experimental samples. Under-correction (*i.e.*, failure to fully remove batch effects) or over-correction (*i.e.*, removal of both technical and biological variability) of batch effects will lead to inaccurate dataset integration and label transfer.

This study aims to improve data integration and annotation results by involving human users and utilizing their feedback to better distinguish batch effects from biologically meaningful variation.

### Data Abstraction.

As shown in Fig. 2A, single-cell transcriptomics data can be represented by a matrix *G* ∈ *R^g×n^* that records the amount of RNA corresponding to *g* genes (rows) detected in *n* cells (columns). Cells in the reference dataset have been assigned cell type labels, while cells in the query dataset have not.

To facilitate the analysis of this high-dimensional data, researchers typically map the original data into a low-dimensional embedding space (*Z_r_ ∈ R^d×n^*, *d* < *g*), which can be used to cluster similar cells. The goal of reference-based cell annotation is to create a joint embedding space where the same types of cells in the reference dataset and the query dataset are well aligned (Fig. 2B-C).

## 3 RELATED WORK

In this section, we summarize the related literature about visualization and integration of single-cell data and visualization in interactive machine learning.

### 3.1 Single-Cell Data Visualization

Interactive visualization techniques are widely used in analyzing molecular measurements in single cells.

Most existing methods for single-cell data visualization focus on gene expression data (*e.g.*, single-cell RNA sequencing or scRNA-seq data) represented as a cell-by-gene expression matrix with cell-level metadata [1]. These methods are often generalizable to other single-cell data modalities (*e.g.*, scATAC-seq data can be represented as a cell-by-peak matrix). Cakir *et al.* [8] summarized and compared these works, including Cytosplore [23], CellxGene [9], Pagoda2 [3], UCSC Cell Browser [59], and ASAP [17]. These tools display dimensionally-reduced single-cell data [7] and support common tasks, including *understanding cell clusters, inspecting the expression magnitude of certain genes* and *annotating cell selections*. Driven by these tasks, most visualization tools feature dimensionality reduction (DR) scatterplot visualizations in their largest, main views. Tools tailored for single-cell data often additionally support expression heatmap visualization, genomic sequence visualization, biomedical file formats, experimental metadata, or contextual ontology information [12].

Single-cell technologies are increasingly able to measure multiple modalities (*e.g.*, quantification of both gene expression and chromatin accessibility in the same cells [11]), temporal context [40], and spatial context (*e.g.*, microscopy images with cell segmentations [35] or spot detections [10], or spatially-resolved sequencing measurements [49]). The existing tools, Facetto [30], SpaCeCo [57], ImaCytE [58], and Vitessce [28], support visualization of microscopy images and cell or organelle segmentations, in addition to the aforementioned features for DR and expression matrix visualization.

The visualization techniques used in *Polyphony* take inspiration from and build upon the existing visualizations for analyzing homogeneous single-cell transcriptomic data. We target an under-explored scenario in which users explore and annotate single-cell transcriptomic data by comparing it to well-established reference datasets. We identify the activities required by this scenario and propose new visualizations to support the workflow in which two datasets are jointly analyzed.

### 3.2 Single-Cell Data Integration

Based on related surveys and benchmarks [2, 39, 63], existing computational methods can be categorized into three main groups: anchor-based, linear mixed model (LMM)-based, and conditional variational autoencoder (CVAE)-based.

Anchor-based methods measure cell-cell similarities and identify correspondences between cells across different datasets [4, 21, 47, 60]. For example, Haghverd *et al.* proposed the mutual nearest neighbors (MNNs) [21] method for single-cell batch correction. MNNs are pairs of cell populations from different datasets close to each other in the latent embedding space. After identifying the MNNs, a transformation can be made to align the datasets according to these cells. Seurat V3 [60] expands upon this method and enables users to integrate single-cell measurements across different modalities. These anchors play an important role in integrating different datasets but are usually hidden from end users, making it difficult for users to understand the model working mechanism and refine model performance.

Another group of works uses LMMs to harmonize single-cell datasets [26, 29]. Symphony [26], a recent work by Kang *et al.*, scales LMM methods to integrate newly produced data with atlas-level references in an acceptable time and memory footprint by compressing each reference into a set of cluster descriptors.

The third group of works uses CVAEs to learn a harmonized joint embedding space [36, 38], as implemented by SCVI [36]. Lotfollahi *et al.* proposed single-cell architectural surgery (scArches), a transfer learning framework that decouples the reference building step from the query data adaption step.

While existing methods demonstrate great performance in many settings, they share certain limitations due to their lack of interactivity and explainability. For example, a novel cell population in the query dataset can be unexpectedly fused to a well-characterized population [26]. Our work aims to incorporate interactivity and explainability in reference-based cell annotation through an interactive machine learning framework. This framework is built upon a CVAE and leverages anchor cell sets, which not only explain the progressive cell annotation process to the user, but also offer users control in the learning process.

### 3.3 Visualization in Interactive Machine Learning

Interactive machine learning (IML) [15] integrates human intelligence with data-intensive computational methods. Various visualization methods have been proposed to reduce user efforts in this process.

Many studies involve users to refine model hyperparameters and working mechanisms. For example, EnsembleMatrix [62] enables users to integrate their knowledge by adjusting the weights of each classifier in an ensemble model. Kapoor *et al.* [27] allow users to adjust the weights of a confusion matrix by assigning a cost for each type of mistake. RetainVis [31] leverages an attention module where users can directly edit attention weights to update the model. Jia *et al.* [25] designed Semantic Navigator, which guides users to steer the model by editing a class-attribute matrix in a zero-shot learning process.

However, model refinement can be challenging for users with limited ML expertise. Therefore, other studies take a different approach, allowing users to inspect and label instances as a way to interact with the ML model [14, 16, 66]. For example, ProtoSteer [41] asks users to edit a list of representative examples from the training data, called prototypes, to steer a deep sequence model [42]. However, biologists conduct population-level analysis using statistical methods on single-cell data. Labeling each individual data instance (*i.e.*, cell) is not feasible.

In this work, we propose an anchor-based approach. Users specify *anchors*, analogous cell populations across the two datasets, to integrate the datasets and transfer the labels from the reference to the query cells.

## 4 Informing the Design

This section presents our collaboration with the domain experts during the design process and the analysis tasks that guide the system design.

### 4.1 Design Process

This study is primarily guided by the Nested Model for visualization design and validation proposed by Munzner [43] and the nine-stage design study framework proposed by Sedlmair *et al.* [52], with focus on the discovery, design, and implementation steps.

We closely collaborated with seven domain experts (**E1-E7**, four females, three males; six postdoctoral researchers and one assistant professor) in single-cell analysis throughout the system design and development process. All experts are experienced in single-cell analysis and have basic knowledge of machine learning methods. None of the experts are authors of this paper. Multiple rounds of expert interviews were conducted at different stages of the study to understand the domain problem and data, clarify analysis tasks, iterate upon the design, and test the prototype system. Finally, *Polyphony* was evaluated with two experts specializing in single-cell transcriptomics data analysis.

### 4.2 Analysis Tasks

Based on the feedback from experts, we summarize four tasks (T1-T4) that users commonly conduct in reference-based single-cell analysis and two tasks (T5-T6) that are desired by users but not well supported in existing tools. While this study focuses on reference-based single-cell analysis, we believe the reported analysis needs and tasks are also insightful for general ML-assisted single-cell analysis.

#### T1 Gain an overview of the joint embedding space.

Experts usually start their analysis with an overview of the cell embedding space, which is typically mapped into a 2D coordinate system using dimensionality reduction methods (**E1-E7**). This overview enables a preliminary assessment of the *clustering quality* (*i.e.*, whether cell type clusters are easily distinguishable) and the *cell integration quality* (*i.e.*, how the cells from different datasets are fused). The annotation results are trustworthy only when the cells are well clustered and integrated under the latent space.

#### T2 Understand cell correspondence across datasets.

After gaining an overview of the embedding space, experts expect to further explore the cell correspondences across the datasets (*i.e.*, which query clusters are nearby which reference cell types, raised by **E5**). The correspondence helps experts understand how their data is mapped onto the reference and suggests how the reference labels might be used to annotate their data.

#### T3 Identify potential unique cell populations.

Experts are generally interested in identifying unique cell populations that appear in the query dataset but not the reference. In specific, when such cell populations are present, experts expect to select query cell clusters that do not overlap with well-characterized cell types for further inspection (suggested by **E2**). In some cases, experts have unique prior knowledge of certain genes, such as cell marker genes that distinguish cell types that have been poorly defined in the literature. They expect to evaluate marker gene expression magnitude to verify and refine the cell population selection (suggested by **E3**).

#### T4 Understand cell identities.

The analysis ends with a full list of annotated cell types. For each cell cluster, experts not only record the cell type label but also the biomarkers (*i.e.*, genes that are differentially expressed among clusters and define cell types). These biomarkers are often related to cell morphology and physiology and play an important role in downstream analysis. Typical machine learning models for cell type annotation only produce cell type labels. Experts (**E2, E3, E6**) expressed their need to identify biomarkers through separate statistical analyses.

#### T5 Understand the integration process.

While ML-based annotation methods enable efficient analyses, the computational process is often opaque to users. Experts expressed the need to understand how each step of the data integration process changes their data. For example, **E6** mentioned that they usually investigate how the clusters in the embedding space change before and after data integration. Importantly, the analysis goal is to gain a deep understanding about the data. Experts wish for a tool that is, *“more than just giving me an answer”* (**E4**).

#### T6 Refine the model to align human knowledge.

The annotation results are not always satisfactory, where the model may under-correct or over-correct the dataset. Instead of checking and manipulating the annotation results one by one, an alternative method is to correct the model systemically, as suggested by **E5**. Enabling users to examine, validate, and refine the model can increase user trust, improve their understanding about the data, and generate more accurate annotations [55].

## 5 Interactive Transfer Learning

We propose an ITL framework that integrates and annotates single-cell query datasets into reference datasets under human supervision. In this section, we first provide an overview of this framework and then introduce the computational method.

### 5.1 Overview

The ITL process (Fig. 3) includes four steps. First, the model compares the two datasets and recommends *anchors* to users (Fig. 3A). Afterwards, users inspect and update these *anchors* according to their domain knowledge (Fig. 3B). The model is then fine-tuned using the user-confirmed *anchors* (Fig. 3C), updating the annotations and embedding for the query dataset (Fig. 3D). The model then recommends new *anchors* based on the updated embedding space. These four steps repeat until the user is satisfied with the integration and decides to apply the predictions to annotate the remaining cells.

The following sections introduce the three major computational components in the framework: model setup, anchor recommendation, and model fine-tuning.

### 5.2 Model Setup

The proposed framework is built upon scArches (single-cell architectural surgery) [37], a transfer learning framework using conditional variational autoencoders (CVAEs) [56] for single-cell data integration. We use SCVI [36] as the backbone model because it is a commonly-used and well-performing single-cell data integration and representation learning method [39].

A CVAE model can generate latent embeddings for the input data where the effects of condition labels are regressed out. In the framework inherited from scArches, such condition labels are used to index batch effects (*e.g.*, sequencing technologies, experiment laboratories, patients, or a combination of these categorical variables). The CVAE model is first trained on the reference dataset and then adapted to the query dataset by adding a conditional node to index batches in the query dataset (Fig. 3C). The parameters in the original model are fixed while the weights related to the new conditional node are learned on the query dataset. As a result, the CVAE model can generate embeddings for the query dataset while removing the batch effects between reference and query. Based on the latent representation learned by the CVAE model, a cell type classifier is trained on the reference dataset to identify the query cell types. We choose a k-nearest-neighbor (kNN) classifier since previous studies [26] illustrate that kNN classifiers can achieve the highest performance while remaining interpretable in this setting.

As with other end-to-end reference-based annotation methods, the CVAE model can potentially over-correct and confuse biological variability with batch effects. Our ITL framework aims to address this issue through a progressive integration method shepherded by users. We set up the CVAE model similar to the settings in scArches to provide a warm start. This model is then refined based on user feedback about the recommended anchors.

### 5.3 Anchor Recommendation

An integration anchor is a pair of cell sets (one from each dataset: query and reference) that share biological similarities and should be proximal in the joint embedding space. Many anchor-based techniques have been proposed to integrate single-cell datasets and remove batch effects [4, 21, 26, 29, 64]. However, the identification of anchors is typically based on a statistical assumption between datasets, which may be invalid in certain scenarios (*e.g.*, the query dataset has a novel cell type). To address this issue, we generate anchors in a progressive manner and involve humans to validate and refine them.

Aiming for scalability to large reference datasets and low time-cost in the interactive system, we adopt the efficient algorithm from Symphony [26], the state-of-the-art atlas-level integration method, to build the anchor recommender. The algorithm contains two steps: *reference building* and *query cell mapping*. The reference building step facilitates scalability by compressing reference datasets into referential elements. Specifically, this step generates a description for each cluster in the reference dataset, including the cluster centers and the number of cells within. We compute this description using the Harmony algorithm [29]. The query cell mapping step aims to find anchors by calculating the assignment probability of each query cell to each cluster. Query cells with high assignment confidence are mapped to the query set of corresponding anchors.

The recommended anchors are represented to the users in a visual interface (Sect. 6), where users can inspect and interact with them through a set of interactive visualizations.

### 5.4 Model Fine-Tuning

We fine-tune the CVAE model based on users’ feedback about the recommended anchors. First, we “correct” the embedding of the user-confirmed anchors by mapping them to the corresponding reference cluster. Inspired by Symphony’s correction algorithm, we use a linear mixed model to update the query cell embeddings by considering only the corrections of the most likely cluster, which users have verified. Then, we fine-tune the model under a semi-supervised setting. Specifically, we expect that the generated latent embeddings of the anchor query cells match the corrected embeddings. Therefore, we add a penalization term in the loss function to measure the distance between the generated and the corrected embeddings for each cell.

## 6 Polyphony

In this section, we introduce the visualization and interaction designs in *Polyphony*. We first provide an overview and briefly introduce an expected analysis workflow. Then, we introduce the design details, rationales, and feedback received from experts during the design process.

**Fig. 1.**
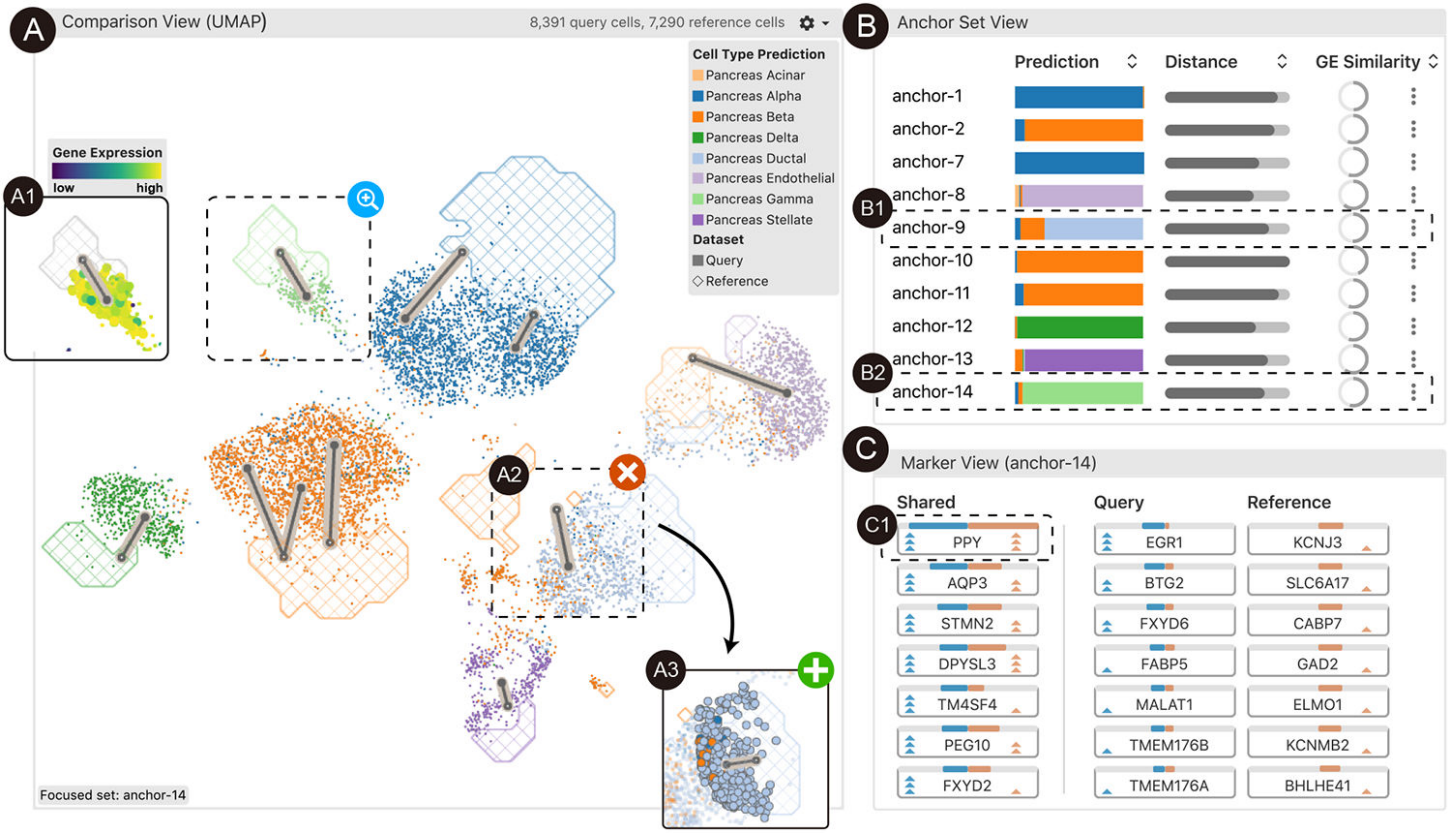
The interface of *Polyphony* contains three views: the *comparison view* (A), the *anchor set view* (B), and the *marker view* (C). The *comparison view* provides an overview of the joint embedding space, and offers users interactions to inspect (A1), delete (A2), and add (A3) anchors. The *anchor set view* orders the anchors in a table, supporting inspection and comparison of different anchors (B1-2). The *marker view* shows the significant genes (C1) for the query and reference cells from a focal anchor.

**Fig. 2.**
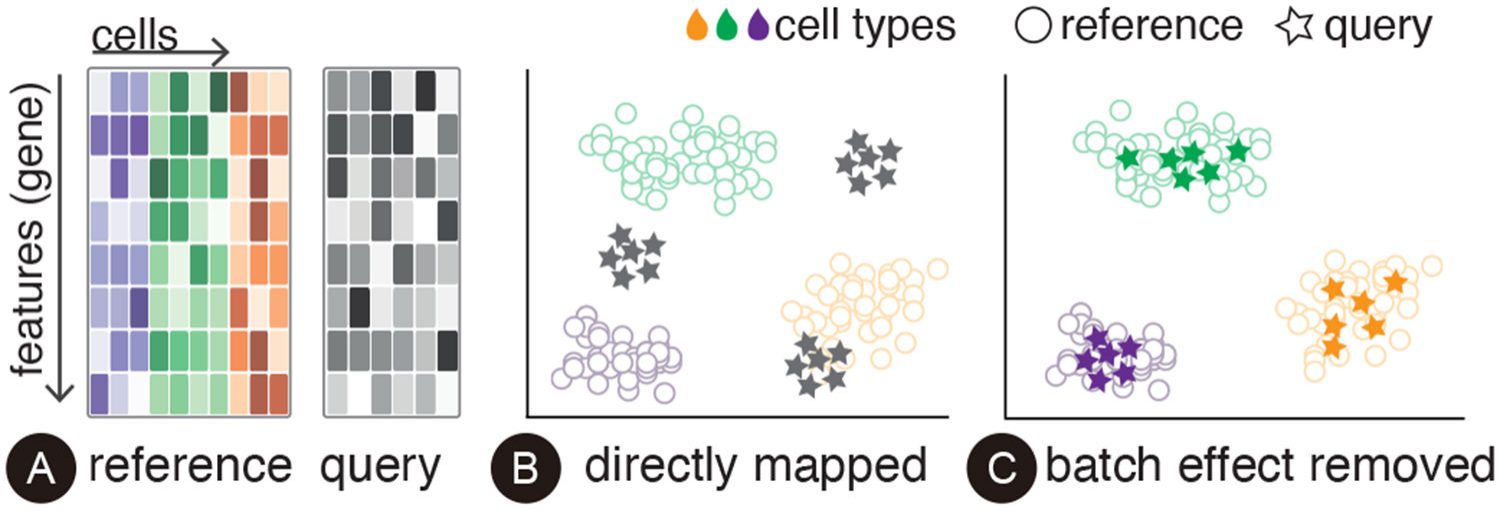
(A) Single-cell transcriptomics data is represented by a matrix that records the amount of RNA corresponding to each gene (row) detected in each cell (column). Batch effects appear in data collected from different studies and must be removed to allow an integrative analysis (B, C).

**Fig. 3.**
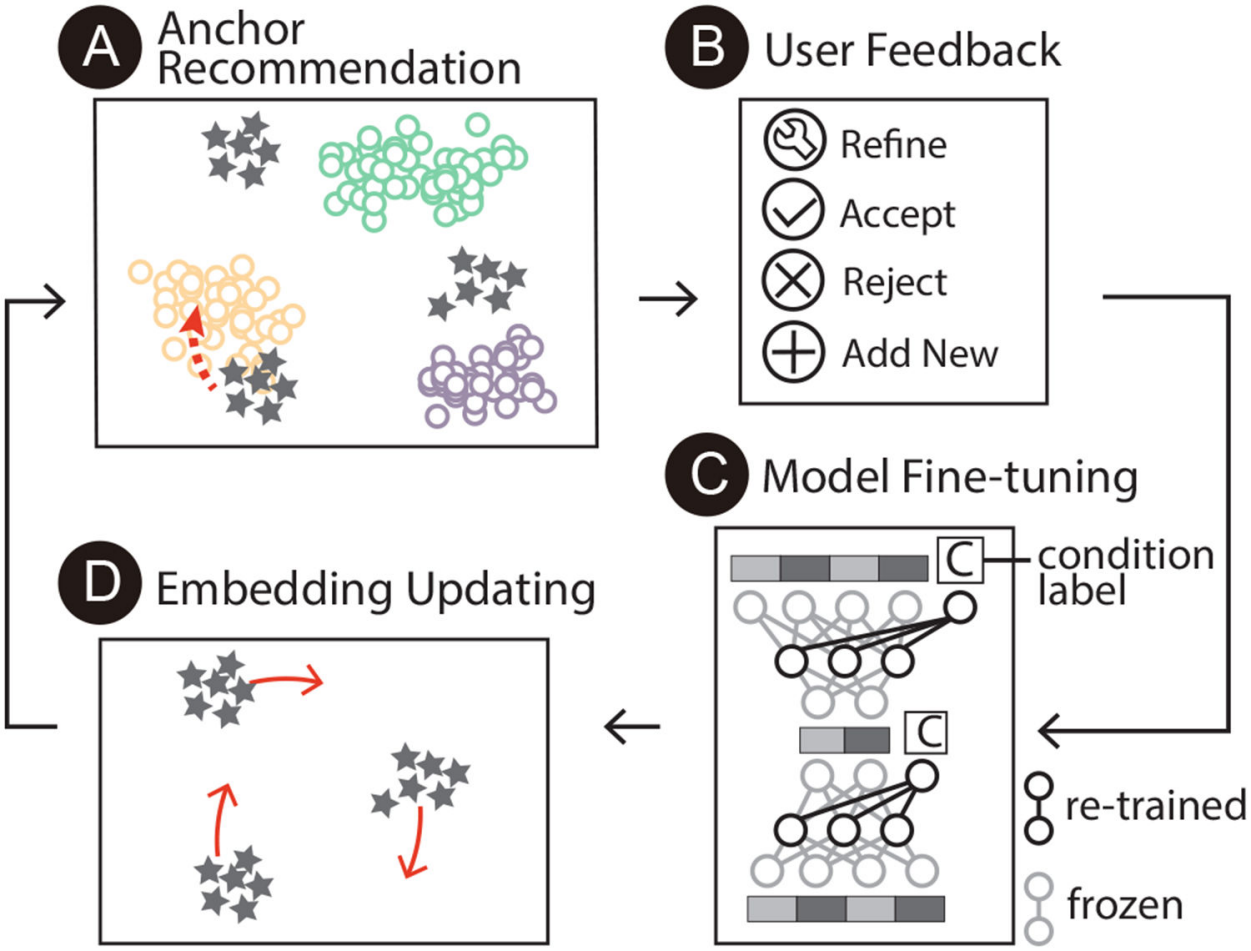
The proposed interactive transfer learning framework includes four key steps: anchor recommendation, user feedback, model fine-tuning, and embedding updating.

The interface of *Polyphony* is composed of three coordinated views, namely the *comparison view*, the *anchor set view*, and the *marker view*. The *comparison view* (Fig. 1A) offers an overview of the embedding spaces (T1), enabling users to understand the distributions of cells and identify cell populations of interest (T3). The *anchor set view* (Fig. 1B) shows statistical information about each anchor (T2). The *marker view* (Fig. 1C) displays differential gene expression analysis results for a focal anchor (T4). These views support interactions that enable users to communicate feedback to the model via anchors (T5, T6).

### Workflow.

A typical workflow starts with the *comparison view*, where the user first understands the quality of the data integration. The user may also find some interesting cell populations as candidate anchors and select them for further inspection. In the *anchor set view*, the user checks the anchors that have either been recommended by the system or selected by themselves. At this stage, the user may directly delete low-quality anchors and confirm promising anchors according to the high-level statistical information. In some cases, the user will check the gene expression values of the anchor cells to make the decision. In the *marker view*, the user inspects and compares the significant genes in both the query cell set and the reference cell set of the anchor. For genes of interest, the user may select each gene and check the expression distribution in the *comparison view*. Finally, after confirming several anchors, the user submits their feedback to the model, prompting fine-tuning and embedding updates. After multiple rounds of model updates, the data integration will have been improved by taking into account human domain knowledge.

### 6.1 Comparison View

**Fig. 4.**
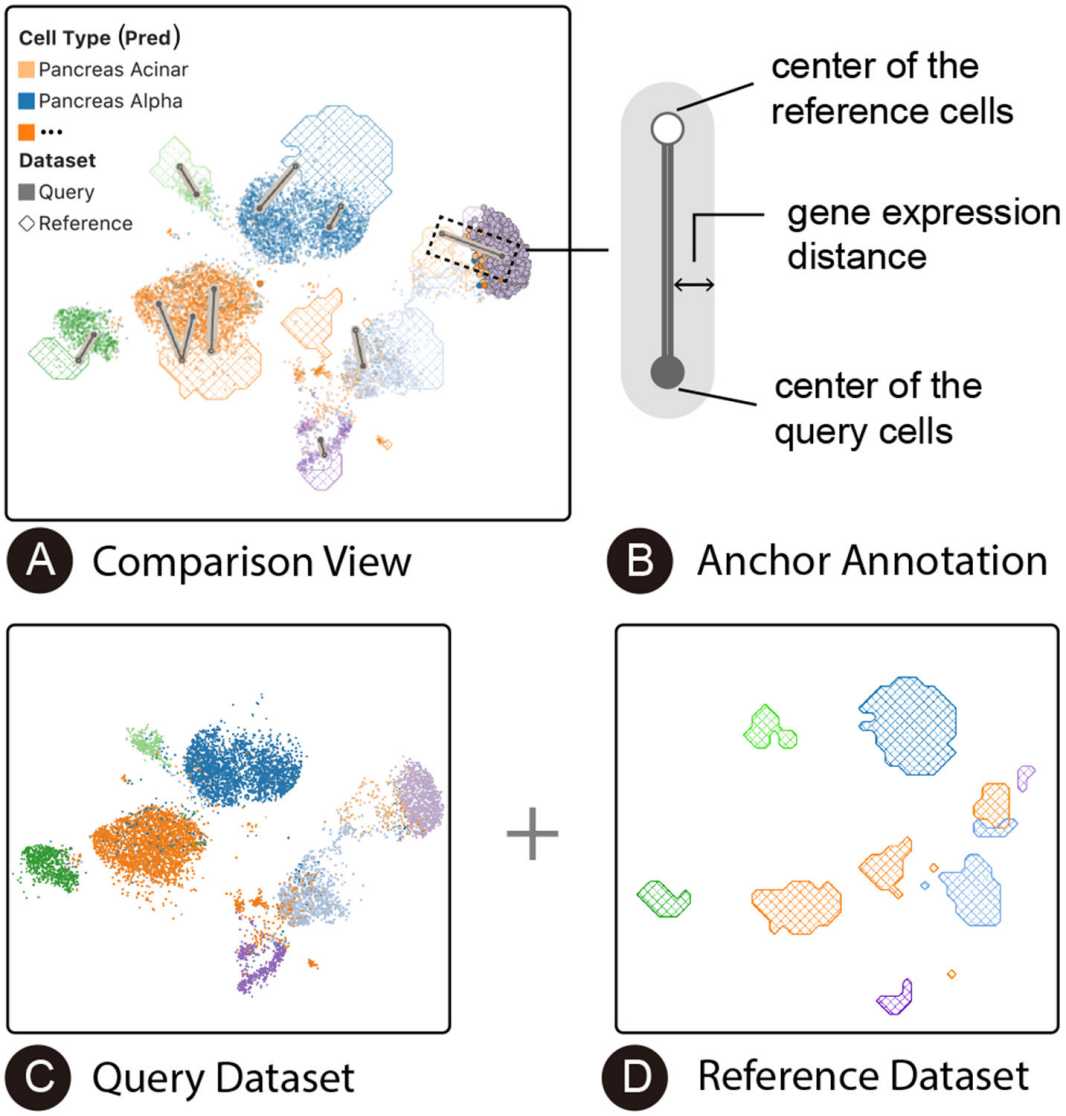
(A) Visualization designs in the *comparison view*. We use different encodings for the query (C) and the reference (D) datasets. The anchor annotations (B) encode the gene expression distances between the query cells and the reference cells, helping users to better understand the integration quality.

The *comparison view* (Fig. 4A) presents both the query and the reference datasets along with different properties of the cells (*e.g.*, cell type labels or predictions, dataset membership, and expression magnitude of selected genes) and their geometry structures in the latent space. This view helps users obtain an overview of the embedding space, evaluate the integration quality (T1), and identify cell populations of interest for further examination (T3). Further, the anchor visualization (Fig. 4B) in the embedding space provides an intuitive approach for users to understand the cell correspondence across datasets (T2).

#### Hybrid design.

We use different visual representations to encode query (Fig. 4C) and reference cells (Fig. 4D) in the same view. For the query cells, we use a scatterplot to provide detailed information about these cells and offer flexible selections. The color of each point encodes the predicted cell type. For the reference cells, we first calculate the density of each type of cell across a grid (with an adjustable grid size) in the projected space. Then, we draw contours to outline dense grid regions according to a unified density threshold adjustable by users. The contour areas are textured to distinguish them from the query cell scatterplot points.

#### Anchor annotation.

Layered above the hybrid visualization of cells from both datasets, we display anchor annotations to help users understand anchor quality and membership in the embedding space context (T2). The annotation design for an anchor (Fig. 4B) is composed of two parts. The inner part contains two linked dots which indicate the centers of the query (solid dot) and reference (hollow dot) cell sets. The outer part encodes the quality of the anchor using the line width (*e.g.*, a wider line denotes lower quality). As suggested by the experts, we use gene expression (GE) similarity as the anchor quality metric. We define GE similarity as the intersection over the union of the gene sets that express significantly differentially between the anchor cells and the rest of the cells, where the significant genes are selected as the top-100 genes when ranked by the *z*-score under a Wilcoxon test. This design enables users to assess anchor quality and identify anchors to inspect and modify depending on the analysis task at hand.

#### Design rationale.

This visualization is an evolution of an initial design, where we used two additional coordinated scatterplots to support the comparison task: one using color to encode the dataset membership of each cell, another using color to encode cell types (or cell type predictions). Despite being straightforward and intuitive to experts, we found that the users were not able to easily join this information together when many cell types were present (*e.g.*, making it difficult to distinguish well-integrated cell types from poorly-integrated cell types). Distinguishing cell type-specific integration quality is essential in selecting proper anchors (T2). This observation motivated us to design a single-view visualization to encode the dataset and cell type information simultaneously. In doing so, we tested both heatmaps and contour lines to visualize the reference cell density layered below the query cell scatterplot. We found that experts prefer contours because heatmaps not only take longer to render but also are visually overwhelming.

### 6.2 Anchor Set View

The *anchor set view* arranges the anchors in a table (Fig. 1B), where each entry represents an anchor that has either been recommended by the system or created by the user. The recommended anchors denote those cells that the model considers most similar between the query and reference datasets (T2). By interacting with these anchors, users understand the model results (T5) and provide feedback for further model refinement (T6).

For each anchor, the table presents the composition of the cell type predictions, the distance between the pair of sets in the latent space, and the GE similarity (introduced in the previous section). These properties were verified by experts to ensure that they represent the most important descriptive information for an anchor. We use horizontal stacked bar plots to show the proportions of predicted cell types, where the color encodes the cell type, consistent with the *comparison view*. In the distance column, we use bar length to indicate the median distance between cells in the query set and the center of the reference set. In the GE similarity column, we use the sweep angle of an arc within a circle to encode the similarity score (in the range 0 to 1).

The GE similarity scores help users make sense of the differences between the query and reference cells in the gene expression space. A lower similarity score indicates that the two cell groups share very different marker genes, suggesting the anchor should be removed in most cases. In contrast, when the similarity score is high, users may still need to investigate the top differentially expressed genes. Sometimes, two similar cell types may share expressions of all but a few critical marker genes whose expression patterns distinguish the cell types. In such a case, expert knowledge is required to make the judgement.

### 6.3 Marker View

**Fig. 5.**
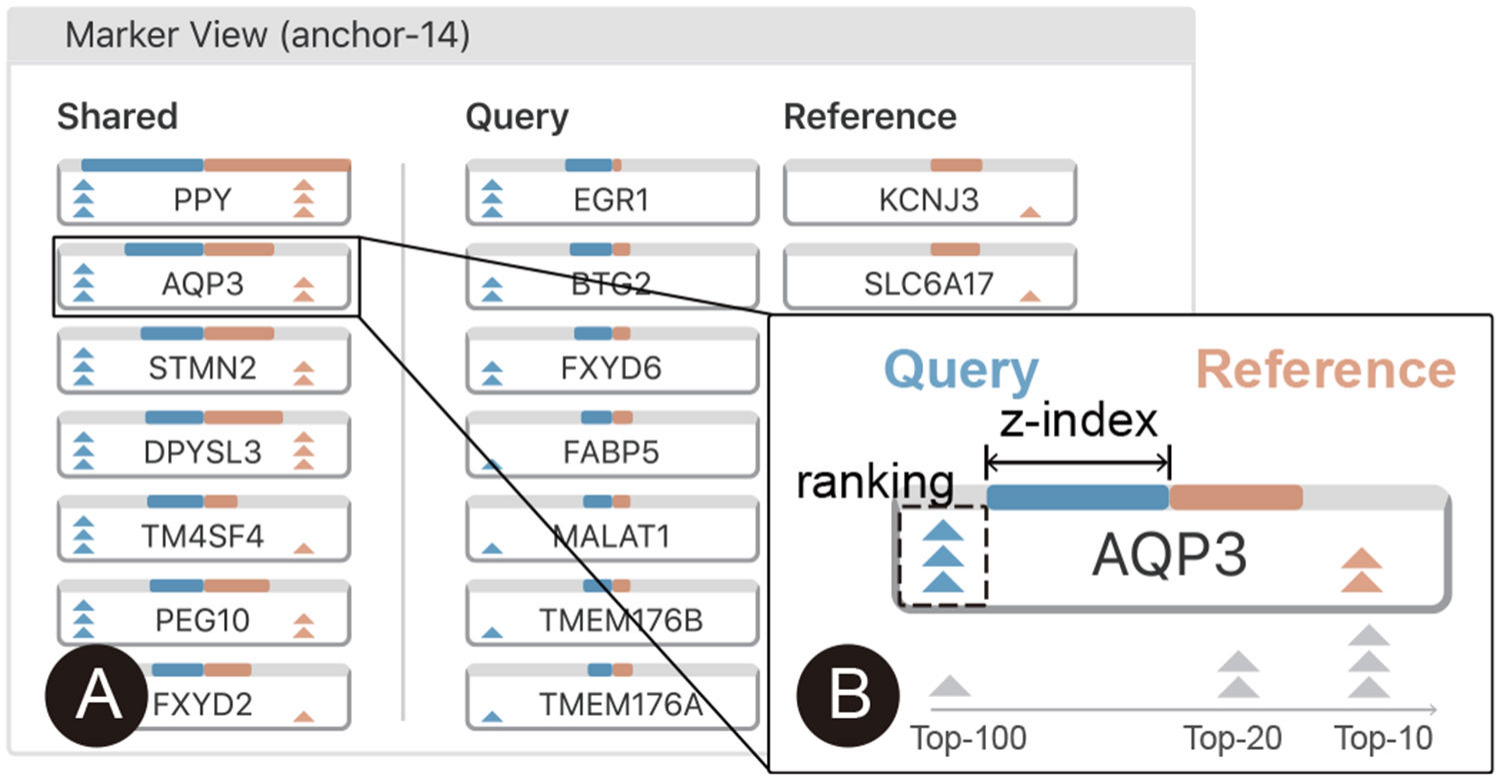
(A) The marker view groups significantly differentially-expressed genes into three columns. (B) The glyph design enables the comparison of gene significance and gene ranking simultaneously.

The *marker view* (Fig. 5A) enables users to conduct gene-level analysis on the significantly differentially-expressed genes (T4).

Suggested by the domain experts, we horizontally divide the interface that lists the significant genes into three columns: significant in both query and reference (“shared”), significant only in query, and significant only in reference. This design helps them distinguish the shared significant genes and those specific to each dataset.

For each gene, we display a glyph (Fig. 5B) to enable the comparison of gene significance (*z*-scores in our case) and gene significance rankings simultaneously in both the query and reference sets. We use two bars with contrasting colors to visualize the *z*-scores of a gene in the query and the reference datasets. A longer bar indicates a higher level of significance. Stacked triangles represent the ranking of a gene in the query dataset (triangles on the left side) and the reference dataset (the ones on the right side). More triangles indicate a higher ranking and imply a more important gene. Three triangles indicate a top-10 ranking. Two or one triangle(s) indicates a top-20 or top-100 ranking, respectively. These thresholds were suggested by the domain experts in our interviews.

To help users better understand the distribution of gene expression in the whole cell population, we coordinate the *marker view* with the *comparison view*. When users click a gene, the color encoding in the *comparison view* will be changed to represent the gene expression magnitude for each cell (Fig. 1A1).

### 6.4 Interacting with Anchors

In this section, we introduce the interactions supported by *Polyphony* to help users explore and update the anchors (T5, T6).

#### Confirm or reject.

The most straightforward interaction with anchors is to directly confirm or reject an anchor. Once an anchor is confirmed, it will be used to fine-tune the back-end model and improve the data integration. In most cases, users need to further examine anchors before they can make a decision confidently, including highlighting anchors, editing anchors, or marking anchors.

#### Highlight.

When users hover over an anchor in the *anchor set view*, the *comparison view* will automatically respond by zooming in and highlighting the corresponding cells and anchor annotations.

#### Edit.

The system provides lasso tools for users to select a group of cells to create a new anchor or update the cells in an existing anchor.

#### Mark.

For anchors that the users are interested in but cannot make a decision confidently, *Polyphony* allows users to affix them to the top of table in the *anchor set* view. Users can keep track of the marked anchors across model iterations and reevaluate their confidence after each iteration.

### 6.5 System Implementation

*Polyphony* contains a front-end user interface for data visualization and a back-end server for data storage, model training, and anchor operations. The front-end is built upon Vitessce [28], a web-based framework for single-cell data visualization. We extend this framework by creating additional reusable components to support reference-based analysis. The back-end is a Python application that relies on Scanpy [65], SCVI-tools [18], scArches [37], PyTorch [45], and scikit-learn [46]. We use Scanpy to preprocess the single-cell data and calculate differential gene expression. The other three packages are used to build the machine learning models. We implement the anchor-powered CVAE model by extending the SCVI model (under the scArches framework) and build the kNN classifiers with scikit-learn. All cell-related data produced in this system (*e.g.*, latent vectors, model predictions, and anchor sets) are compressed and stored in h5ad [65] files allowing users to easily share the results. Finally, we use Flask to develop a server for communicating between the front-end and the back-end.

## 7 Evaluation

We evaluate *Polyphony* from both the algorithmic perspective and the user perspective. In the algorithm evaluation, we conduct quantitative experiments to evaluate the performance of the *Polyphony* model under different anchor selection policies. In user-centered evaluations, we evaluate the utility and usability of the whole system from hypothetical use cases and qualitative feedback from experts.

### Datasets.

We apply two commonly-used benchmark datasets in single-cell transcriptomics data integration: the *Human Pancreas Dataset*, which is constructed by combining results from [5, 20, 33, 44, 53], and *Human Peripheral Blood Mononuclear Cell (PBMC) Dataset* [13]. The Pancreas dataset contains gene expression measurements of 15,681 cells across 9 cell types from human pancreas. We separate the datasets into a reference set (cells generated using a plate-based protocol, *n* = 7290 cells) and a query set (cells generated using a droplet-based protocol, *n* = 8391 cells). The PBMC dataset contains gene expression profiles produced by various sequencing techniques for over 32,300 total cells. We divide them into a reference set (21,573 cells from eight batches profiled by multiple technologies) and a query set (10,727 cells profiled by the 10X Genomics Chromium technology).

### 7.1 Algorithm Evaluation

To study the effectiveness of the *Polyphony* model in improving the quality of single-cell data integration and annotation, we designed and conducted the following experiments.

#### Methodology.

We evaluate the model performance by simulating different strategies that the experts may apply to confirm anchors during the model iteration (*e.g.*, accept most anchors recommended by the model vs. select a few high-quality anchors). To simulate how experts assess anchor quality using their domain knowledge of particular cell types, we estimated the quality of an anchor based on the cell-type consistency between query and reference cells. Specifically, we calculated a normalized entropy value for each anchor based on the ground-truth types of the query and reference cells. We use a threshold *θ* to denote an anchor selection policy (anchors whose normalized entropy values are smaller than *θ* are selected). A lower threshold *θ* indicates a stricter policy where fewer high-quality anchors are selected. And *θ* = 1 indicates that all recommended anchors are selected for model updating.

#### Settings.

In total, we run four experiments under different anchor selection policies (*θ* ∈ {0,0.25,0.5,1}) for each of the two datasets (*i.e.*, Pancreas and PBMC). With *θ* = 0, the model uses no anchors (*i.e.*, under the same settings with scArches [37]). We denote this condition as the baseline. Each experiment starts with a unified warm-up session (100 epochs) where the models initially trained on the reference dataset incompletely adapt to the query dataset. Then we run each experiment with multiple rounds of anchor-involved model updating. We report the results at the fourth round since most valid anchors are already selected after four rounds according to our experiments. We apply two commonly used metrics based on local inverse Simpson’s Index (LISI), iLISI and cLISI [29], to evaluate the integration quality and use the macro-averaged F1 score to evaluate the annotation accuracy. All three scores are normalized from 0 to 1 [39], where higher iLISI, cLISI, and F1 scores indicate better batch-effect removal, cell type separation, and annotation accuracy, respectively. We run each experiment ten times and report the average results and the variance in Table 1.

#### Results.

As shown in Table 1, with both datasets, the *Polyphony* models (*i.e.*, policies where *θ* > 0) outperform the baseline models in single-cell data integration (based on iLISI and cLISI scores) and annotation (based on F1 scores). Besides, models with more confirmed anchors (*i.e.*, a higher *θ*) achieve higher iLISI scores, indicating that the cells between the query set and the reference set are better mixed and batch effects are more sufficiently removed. However, models with more anchors do not necessarily have more accurate annotations. For example, in the Pancreas dataset, the models with the anchor selection threshold *θ* = 0.5 achieve the highest F1 score on average (0.806). And in the PBMC dataset, the highest F1 score (0.621) is achieved under a stricter threshold *θ* = 0.25. We hypothesize that this is due to the confirmation of low-quality anchors, which mix different cell types and reduce the annotation accuracy. The hypothesis is supported by the cLISI scores, where models confirming all anchors (*θ* = 1) achieve lower cLISI scores than the models with stricter policies.

The experiment results confirm the effectiveness of the *Polyphony* model in single-cell data integration and annotation. The results also reveal the importance of making selections of faithful anchors, where expert judgement is required.

### 7.2 User-Centered Evaluation

In this section, we introduce the user-centered evaluation of *Polyphony* with two use cases and qualitative feedback from experts.

#### 7.2.1 Use Case I - Pancreas Dataset Integration

In the first use case, we use the Pancreas dataset. A hypothetical biologist wants to annotate the cells in the query set using the reference cells. However, the technical variation between the two datasets introduces batch effects and prevents direct annotations. We describe how the biologist would use *Polyphony* to overcome this challenge and conduct an integrative analysis.

##### Understand the quality of integration.

First, the user looks at the *comparison view* (Fig. 1A) to understand how the two datasets have been integrated together (T1). The user notices that few cells from the query dataset (represented by scatterplot points) fall within the textured contour areas, where the cells from the reference dataset are most dense. This indicates that batch effects are still present despite an initial round of integration. The user feels that biologically relevant conclusions cannot be obtained until such batch effects are removed.

##### Understand and refine anchor recommendations.

The user then inspects the anchors recommended by the system (T2). The user feels that most of these recommended anchors look reasonable. One exception is “anchor-9”, which seems to link a group of Pancreas Ductal cells (■) from the query dataset to Pancreas Beta cells (■) (Fig. 1A2) in the reference (T5). Referring to the *anchor set view*, the user notices that this anchor contains multiple types of cells (Fig. 1B1), indicating its low quality and thus should be removed (T6). Instead, the user wants to create a more reasonable anchor to integrate Pancreas Ductal cells (■). The user uses the lasso to select a group of such cells near the reference contour (Fig. 1A3) and confirm this newly created anchor.

##### Inspect gene expressions and modify anchor.

The user wants to further understand the gene profiles of the remaining anchors (T4). The user selects “anchor-14” (Fig. 1B2), which is mainly composed of Pancreas Gamma cells (■). From the *marker view*, the user notices that *PPY* is the top-most significant gene for both the query and the reference cells. The user knows that this gene is a widely accepted marker gene for this cell type and anticipates that this anchor will be helpful in integrating the Pancreas Gamma cells (■) from the two datasets together. The user then checks the expression magnitude of *PPY* by clicking on it. The user notices that the anchor cells are colored bright yellow (Fig. 1A1), distinguishing them from the rest of the cells, which confirms the hypothesis. After applying the same strategy to check and confirm other anchors, the user updates the model.

##### Summary.

**Fig. 6.**
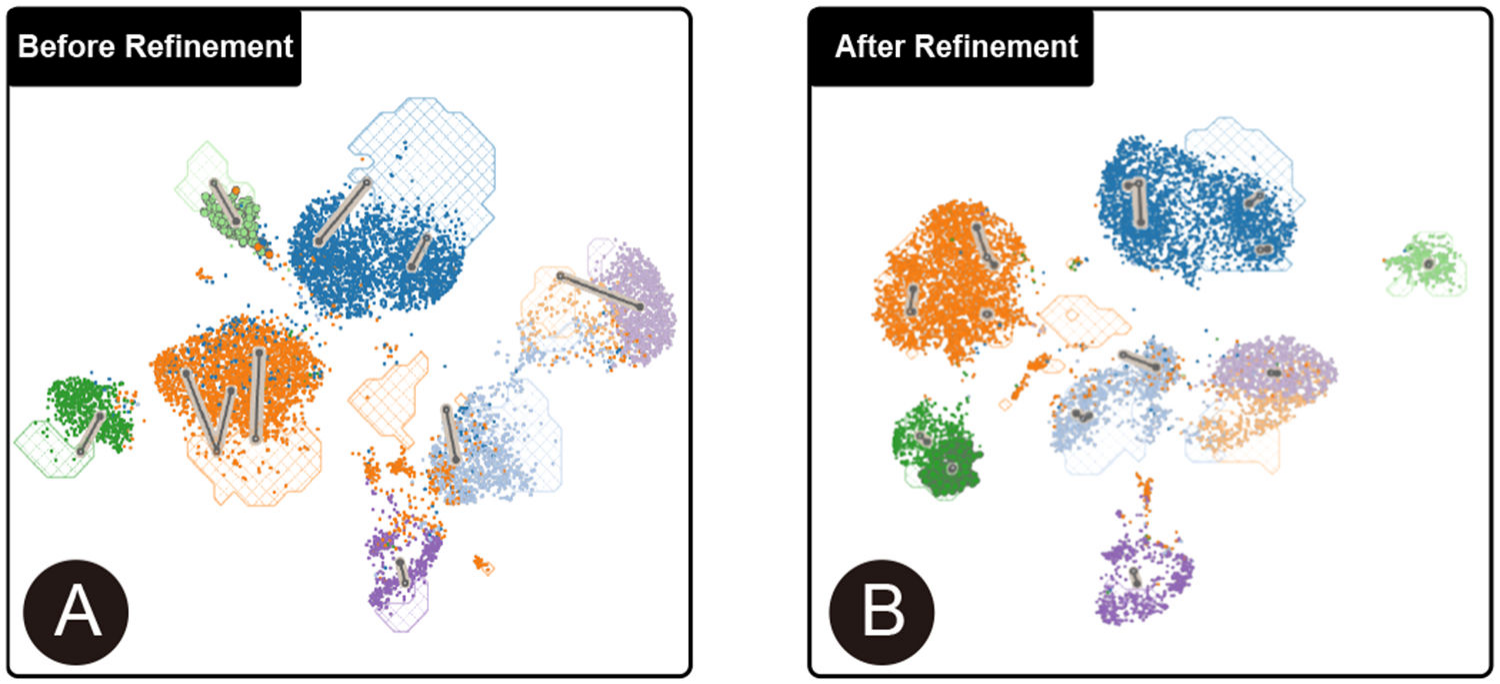
The joint embedding space before (A) and after (B) the integration guided by user-specified anchors.

The model returns illustrate a better integration between the query and the reference datasets (Fig. 6B). Involved in the integration process, the user feels confident about the integration results.

#### 7.2.2 Use Case II - PBMC Dataset Understanding

We consider a more challenging scenario in which the cell types of the query dataset are not fully reflected in the reference dataset (*i.e.*, the query dataset includes novel cell types). We use the PBMC dataset. Following common practices in molecular biology, we simulate the mismatch in cell-type composition between the query and reference by intentionally removing all plasmacytoid dendritic cells (pDCs. 184 cells, 0.8%) from the reference dataset [26]. In this use case, the user is aware of the potential cell composition mismatch between the query and reference. We show how *Polyphony* can be used to distinguish these unseen cells in a reference-based analysis.

##### Bad integration or novel cell population.

**Fig. 7.**
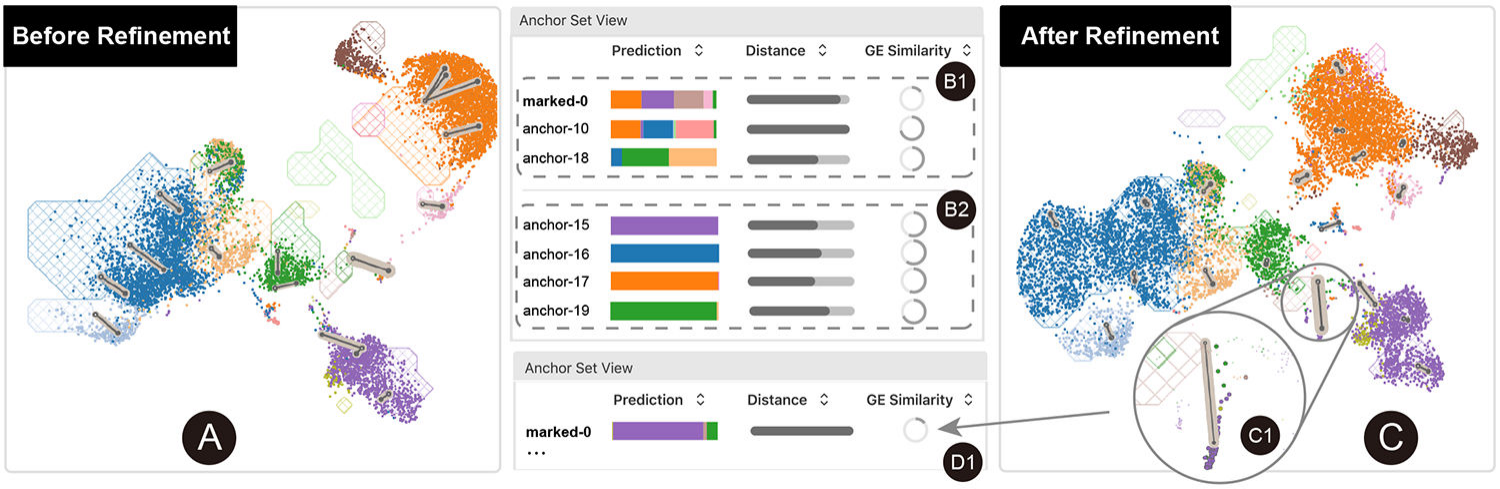
Integrating and discovering unknown cell populations from the PBMC dataset. The user first gains an initial impression of the two datasets (A) and inspects some low-quality anchors (B1), which potentially contain unknown cell populations. Then, the user tries to improve the integration quality by confirming high-quality anchors containing familiar cell types (B2). The updated embedding fuses the two datasets well (C). An exception is an anchor with a wide border in its anchor annotation (C1), corresponding to a previously-marked anchor (D1). After checking its marker genes, the user confirms that these anchor cells belong to pDC, a cell population missing from the reference dataset.

The user first checks the *comparison view* to gain an overall understanding of the two datasets (Fig. 7A) (T1). At a glance, the user notices that multiple cell clusters in the query dataset do not overlap with any reference cluster. Besides, some anchors have thick borders, indicating that batch effects remain (T2). Then, the user turns to the *anchor set* view and finds some “suspicious” clusters with either a very low GE similarity or multiple predicted cell types at high proportions (Fig. 7B1). However, the noise introduced by batch effects makes it hard to distinguish whether the “suspicious” clusters represent novel cell populations or simply misalignments (T3). The user decides to improve the integration quality and return later to investigate the “suspicious” clusters.

##### Improve the integration with anchors.

The user selects anchors with homogeneous cell type prediction composition and high similarity scores (Fig. 7B2). These anchors contain common immune cells, including *CD20*^+^ B cells (■), *CD4*^+^ T cells (■), *CD14*^+^ Monocytes (■), and NK cells (■). The user is familiar with these cell types and thus can make confident decisions about whether to confirm them. After ensuring that the corresponding marker genes are highly expressed in each anchor (T4), the user confirms them (T6). After the model has been updated, the user finds that most query cells are well aligned with the reference in the new latent space (Fig. 7C). It indicates that batch effects have been removed, and most query cells have been mapped into the reference space such that similar cells are nearby.

##### Identify unknown cell populations.

Despite the good alignment of most query cells, the previously-marked anchor “marked-0” stands out with a relatively long distance between the query and the reference cell sets (Fig. 7C1). Most importantly, the query and the reference sets are marked by different genes, as indicated by the low GE similarity (Fig. 7D1). The user feels that the query cells likely belong to a novel cell type. After inspecting the marker genes and referring to related literature and external tools, the user finds the marker genes related to *stimulus response, defense response*, and *immune effector processes*. The user finally confirms that these cells belong to the pDC cell type.

##### Summary.

Through this analysis, the user finds a rare cell population from the query dataset. The finding is supported by the cluster structure in the refined joint embedding space and the statistical evidence of the differentially expressed genes.

#### 7.2.3 Expert Interview

We conducted individual interviews with two experts (**E3, E4**) in a semi-structured way. Since the experts were already familiar with this project from previous interviews, we briefly reminded them about the background and demonstrated *Polyphony* with a showcase using the pancreas dataset. Then, we let the experts explore the system for 30 minutes freely. The participants were encouraged to ask questions during the exploration. We took notes on the system usage and asked the experts the rationale behind their operations upon anchors. Afterward, we collected their feedback on their usage experience, expected usage in their work, and desired improvements.

##### System design.

Both experts agreed that visualization designs in *Polyphony* were intuitive and that the system was easy to use. At first, **E3** expressed confusion that an anchor did not contain all cells in what appeared to be a cluster in the embedding. After explaining that anchors are analogous cell sets across datasets that the model is confident about, they understood. They highly appreciated the flexibility in selecting cell populations of interest and obtaining recommended reference cells. They commented, “*this tool does not just give me an answer but allows me to interact with it*.” **E4** suggested that in their studies, they noticed that existing computational methods for integration sometimes produce undesired results. They highly appreciated that *Polyphony* enables improving the integration through their feedback.

##### Anchor validation.

**E4** confirmed that whether an expert confirms or rejects anchor recommendations highly depends on the expert’s knowledge about the corresponding cell type and marker genes. They commented, “…*if I knew this type of cell and saw related marker genes expressed, I would be pretty sure about the anchor*.” Otherwise, they need to check the literature to make the decision. **E3** expressed similar opinions and suggested that certain types of cells are more likely to be chosen as the anchors by users than others, stating, “…*some cell types are well-understood by biologists, while some are hard to define*.”

##### Application scenarios.

Both experts said that they would use the system to understand how the integration was performed and make further improvements. **E4** suggested that this process can help them to understand unfamiliar cell populations in their data. They described the process as first using the familiar cell populations as anchors to integrate the datasets. Then, they would check how the unfamiliar populations are mapped to the reference for further understanding. From a different angle, **E3** commented that they would like to use the system to conduct comparative studies of cells from different sources. For instance, they might compare cells from different organs or from different patients by dividing them into a treatment group and a control group.

##### Desired improvements.

Both experts are eager to use the system with their own data (with over 100,000 cells). They also wanted the system to contain multiple pre-loaded reference datasets (*e.g.*, cells collected from different organs) to select from. Further, **E3** hoped that the system could enable users to simultaneously select multiple cell populations and perform differential expression analysis within the selected sub-populations of cells. **E4** suggested that the system could also integrate gene set enrichment analysis [61] to provide annotations for genes, which would help experts quickly gain a high-level understanding of the potential functions of the cell populations.

## 8 Discussion

We discuss the impact on the target domain, design implications, limitations of this work, and directions for further improvements.

### 8.1 Impact on Single-Cell Data Analysis

Our system improves the biologist’s workflow in reference-based single-cell data analysis by combining two traditionally separate analysis stages—*understanding* and *integration*—together in an interactive and iterative process. We extend the usage of anchors by supporting user inspection of and interaction with them through tailored visual summaries and interaction designs. Such improvements allow users to gain insights into cell correspondences and interact with the model.

A typical challenge in the joint analysis is to distinguish whether an unexpected integration result is an insight (*e.g.*, novel cell populations) or an artifact (*e.g.*, results from an imperfect integration algorithm). In current practice, biologists run an integration algorithm to gain a rough result and manually check the results on cell clusters of interest. When they find unexpected results (*e.g.*, remaining batch effects), there is limited support for model refinement. Some biologists may choose to run the integration algorithm with different parameters multiple times. Others may abandon the results and refer to different integration algorithms. Both approaches are inefficient. *Polyphony* aims to improve this workflow by providing controllability and interactivity for an integration model. Specifically, our system enables users to steer the integration process via anchors based on their observation of the integration results and their domain knowledge about cell types. Observing these cells and interactively steering the model allows users to conduct the joint analysis with much-improved efficiency.

### 8.2 Design Considerations and Implications

#### Support human-model communication with anchors.

In the proposed framework, anchors serve as a medium for human-model communication and allow experts to monitor and steer the model. We use anchors based on the following considerations. First, case-based reasoning (or analogical reasoning) [50] is one of the most common human reasoning procedures. Thus, showing concrete examples (*i.e.*, anchors in this work) rather than model parameters or metrics helps experts understand and improve integration results in a more user-friendly way. Second, using analogous cell populations to “anchor” the two datasets is similar to biologists’ problem-solving process, where they use familiar cell populations to ensure the integration quality and make further inferences about the unknown populations. Third, from a technical perspective, anchors allow the model to be refined through semi-supervised learning, which helps to improve the integration quality with human knowledge. According to our evaluation, this approach is intuitive to experts and can greatly improve the model performance.

#### Compare embedding spaces.

Most existing embedding-space comparison techniques assume that the correspondences between the two embedding spaces are clear [6, 54]. For example, they compare the embeddings of the same group of words generated by different embedding algorithms. In this work, we focus on a different problem setting where the two datasets have different embeddings (caused by batch effects) and contain different elements (*i.e.*, the correspondences between the two datasets are ambiguous). Specially, we use links to indicate anchors and thus visually enhance users’ perception of the correspondences (Fig. 4D). Furthermore, considering the ambiguity of correspondences and the risks of leading users to integrate two different cell populations incorrectly, we encode the GE similarity using the width of the lines. This helps users to quickly find potential low-quality anchors, which may provide insights into novel cell populations.

### 8.3 Limitations and Future Work

#### Scale to large datasets.

*Polyphony* currently supports the analysis of moderately-sized real-world datasets containing tens of thousands of cells. We tested *Polyphony* using three datasets. Two are moderately-sized datasets that are introduced in Sect. 7 and one is a large-scale dataset containing 274,346 immune cells from patients with COVID-19 (154,723 reference cells and 62,469 query cells) [34]. The model updates (with 50 epochs) take less than a minute for the two moderately-sized datasets and take about four minutes for the COVID dataset. These experiments were conducted on an Amazon server with an NVIDIA Tesla K80 GPU.

While the visualization front-end in *Polyphony* can display hundreds of thousands of cells with smooth interactions, preprocessing steps (*e.g.*, computing anchor centroids) are a bottleneck (three minutes for 200,000 cells). This can be reduced by moving steps to the back end, facilitating use with the largest available references (*e.g.*, Azimuth references, which range from 76,533 to 584,884 cells [22]).

#### Select meaningful reference dataset.

*Polyphony* assumes a high-quality reference dataset and focuses on transferring labels from the reference to annotate query datasets. However, users may inadvertently use a low-quality reference (*e.g.*, wrong labels, mismatched cells), which can severely undermine the annotation results. In the future, we intend to learn how reference quality influences system usage and make improvements in two areas. We plan to provide pre-loaded high-quality reference datasets in *Polyphony*. To achieve this, we will deploy the system to a cloud server, making it accessible to biologists worldwide, and prepare a gallery of pre-loaded reference datasets.

#### Support multi-modal and spatial omics data.

In this work, we focus on the use of *Polyphony* with single-cell transcriptomics datasets. In recent years, multi-modal omics measurement techniques have enabled biologists to simultaneously measure DNA, RNA, and protein abundance and accessibility in the same cells, often with spatial context, enabling a more comprehensive understanding of biological processes. In future work, we plan to extend the anchor-based framework to support multi-modal and spatial omics data. A promising direction is to leverage multi-modal models, such as totalVI [19], to learn joint embeddings for which visualizations can be designed to support group-level comparisons across different modalities.

## 9 Conclusion

In this work, we propose *Polyphony*, an interactive transfer learning (ITL) framework that helps biologists integrate and jointly analyze single-cell data with annotated references. The framework leverages *anchors*, analogous cell populations across datasets, to support interactions between humans and machines. We develop an interface through an iterative design process to support user understanding of the integration quality and enable integration improvements through a set of operations on *anchors*. We demonstrate the usefulness and effectiveness of this approach through quantitative experiments, two use cases, and interviews with two biologists. The results reveal that the anchor-based approach offers users an efficient way to interact with machine learning models for understanding and improving single-cell data integration results. Finally, we summarize the lessons learned from this study to inspire future studies on reference-based single-cell analysis and human-model interactions.

## Supplementary Material

Supplementary Video

## Figures and Tables

**Table 1.** The model performance under different anchor selection policies.

	Pancreas Dataset (*n* = 15681)			PBMC Dataset (*n* = 32300)		
	iLISI	cLISI	F1 Macro	iLISI	cLISI	F1 Macro
Baseline	0.390 ± 0.020	0.9944 ± 0.0005	0.760 ± 0.011	0.230 ±0.015	0.9906 ± 0.0005	0.556 ± 0.007
Polyphony (*θ* = 0.25)	0.530 ± 0.021	**0.9960** ± **0.0003**	0.784 ± 0.026	0.442 ± 0.026	**0.9929** ± **0.0006**	**0.621** ± **0.036**
Polyphony (*θ* = 0.5)	0.549 ± 0.027	**0.9960** ± **0.0004**	**0.806** ± **0.015**	0.459 ± 0.019	0.9925 ± 0.0006	0.610 ± 0.035
Polyphony (*θ* = 1)	**0.562** ± **0.027**	0.9959 ± 0.0004	0.799 ± 0.015	**0.473** ± **0.013**	0.9920 ± 0.0006	0.582 ± 0.014

LISI: local inverse Simpson’s Index; iLISI: integration LISI; cLISI: cell-type LISI [29]. PBMC: Peripheral Blood Mononuclear Cell [13].
